# In Vitro Mitral Valve Model with Unrestricted Ventricular Access: Using Vacuum to Close the Valve and Enable Static Trans-Mitral Pressure

**DOI:** 10.1007/s12265-021-10199-5

**Published:** 2022-01-06

**Authors:** Sam E. Stephens, Alexander J. Kammien, Jacob C. Paris, Alexis P. Applequist, Neil B. Ingels, Hanna K. Jensen, Drew E. Rodgers, Charles R. Cole, Jonathan F. Wenk, Morten O. Jensen

**Affiliations:** 1 Department of Biomedical Engineering, University of Arkansas, Fayetteville, AR USA; 2 Department of Surgery, University of Arkansas for Medical Sciences, Fayetteville, AR USA; 3 Department of Anesthesiology, Washington Regional Medical Center, Fayetteville, AR USA; 4 Department of Cardiovascular Surgery, Washington Regional Medical Center, Fayetteville, AR USA; 5 Department of Mechanical Engineering, University of Kentucky, Lexington, KY USA

**Keywords:** Mitral valve, In vitro, Surgery, Papillary muscles, Chordae tendineae, Mitral annulus

## Abstract

Current in vitro models of the left heart establish the pressure difference required to close the mitral valve by sealing and pressurizing the ventricular side of the valve, limiting important access to the subvalvular apparatus. This paper describes and evaluates a system that establishes physiological pressure differences across the valve using vacuum on the atrial side. The subvalvular apparatus is open to atmospheric pressure and accessible by tools and sensors, establishing a novel technique for experimentation on atrioventricular valves. Porcine mitral valves were excised and closed by vacuum within the atrial chamber. Images were used to document and analyze closure of the leaflets. Papillary muscle force and regurgitant flow rate were measured to be 4.07 N at 120 mmHg and approximately 12.1 ml/s respectively, both of which are within clinically relevant ranges. The relative ease of these measurements demonstrates the usefulness of improved ventricular access at peak pressure/force closure.

**Graphical abstract:**

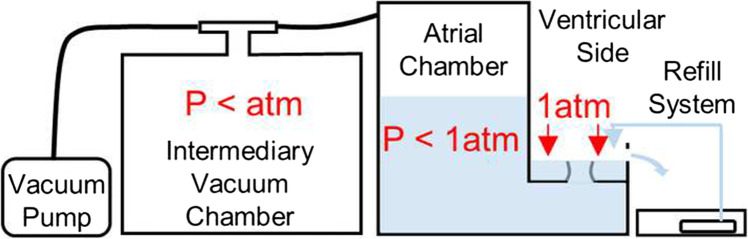

## Introduction

The mitral valve (MV) supports the unidirectional pumping of blood through the heart by allowing blood flow into the left ventricle during diastole and precluding retrograde flow into the left atrium during systole. Mitral regurgitation, the backward flow of blood through the valve into the left atrium, affects 1.7% of the general population and 9.3% of people over the age of 75 in the USA [1]. Leaflet coaptation geometry, annular shape and size, papillary muscle (PM) position and orientation, and chordal force distribution are critical to proper valve function, and even slight disruptions can result in impairment. For example, PM positioning and annular geometry have been shown to affect leaflet strain and chordal force distribution on the leaflets [2–4]. Displacement of PMs and dilation of the annulus can cause significant mitral regurgitation by inhibiting leaflet closure and interrupting the geometry of leaflet coaptation [5].

Experimental work with the MV apparatus in vivo, in vitro and in silico has, for several decades, added to the knowledge of MV mechanics and improved the efficacy of treatment for mitral regurgitation. In vitro models are highly relevant in providing access to the valve and the ability to isolate certain conditions. These models can also serve to providing boundary conditions for computational work, in which force-validated input has proven critical [6–9]. In vitro studies require the establishment and maintenance of a trans-valvular pressure difference to close the valve in dynamic and static systems [10–14]. These gradients are commonly established by increasing pressure on the ventricular side of the valve relative to the atrial pressure. Maintaining a constant pressure difference typically involves two chambers, representing the left ventricle and left atrium, that are connected by the MV [10]. Because the ventricular chamber must be sealed to hold pressure, it inhibits outside access to the subvalvular apparatus. Previous successful models have been both dynamic and static [2, 10–17]. Using static models removes the potential limitation of fluid density and viscosity inconsistency between experimental in vitro and in vivo fluid dynamic environments. In addition, static models allow the system to “freeze” the valve in the most critical phase of the cardiac cycle where the valve is closed under peak trans-mitral pressure.

Establishing trans-mitral pressure differences with negative pressure—relative to atmospheric—on the atrial side of the valve is unique and will leave the ventricular side unpressurized and accessible with atmospheric pressure. To the best of our knowledge, this is the first demonstration of this novel technique for in vitro assessment of function for any of the atrioventricular valves. The primary aim of this study was to develop and evaluate an in vitro testing system that utilizes negative vacuum pressure to close the MV and maintain the systolic load in a static configuration. As a result, systolic access to vital subvalvular structures, such as the PMs and chordae tendineae (CT), is greatly increased, improving the ability to investigate critical valvular changes implicated in MV disease and regurgitation. A demonstration of the utility of the setup is provided by the measurement of total PM force and regurgitant flow under static, systolic loading conditions, which is compared to clinically relevant in vivo and previous in vitro dynamic systems. The system is intended for capturing the fully closed valve at a positive trans-ventricular-atrial pressure and various absolute systolic pressures.

## Materials and Methods

**Fig. 1:**
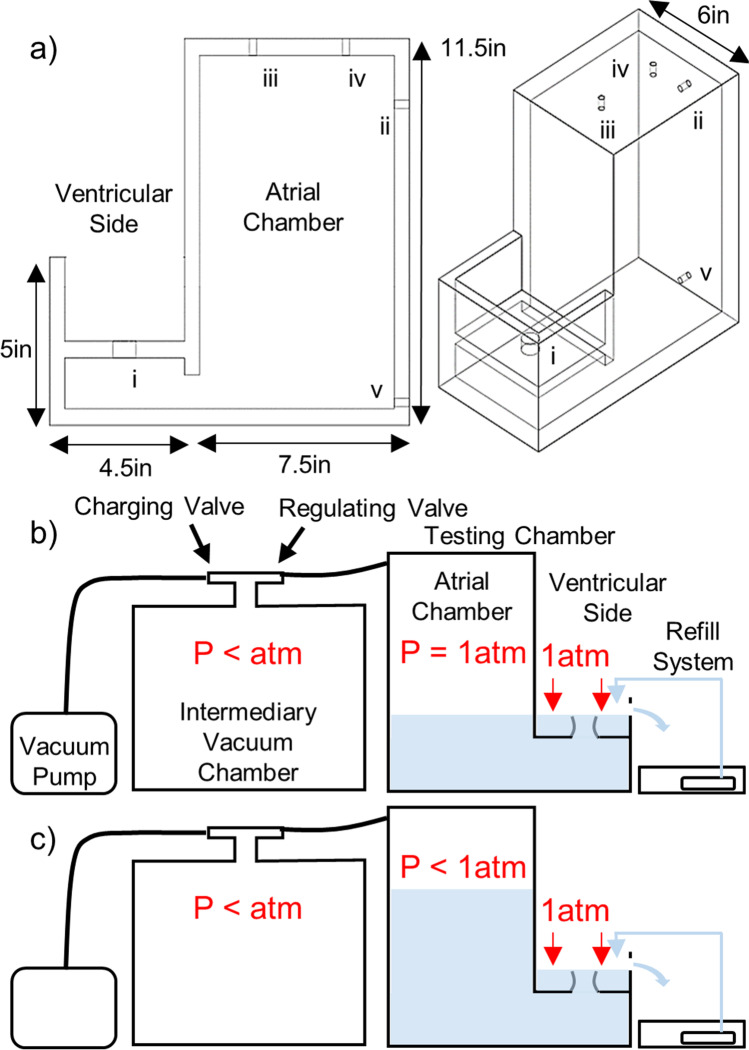
Schematic representation of the left heart *in vitro* system, showing **a** side view (left) and isometric view (right) of the left heart model. Important components: (i) ventricular/atrial orifice, (ii) vacuum system input, (iii) vacuum gauge input, (iv) vacuum release valve, and (v) drain valve. Also shown is the testing system immediately before use of the vacuum system (**b**) and after valve closure (**c**). The white space inside the testing chamber represents the atrial-chamber airspace while the blue area represents water

The in vitro static left heart vacuum-based system has four primary components: vacuum generation, left heart model, valve mounting system, and refill system (Fig. 1). The left heart model has two compartments representing the left atrium and left ventricle. The atrial chamber interfaces with the vacuum system, allowing for the establishment of negative pressure within the atrial chamber while the ventricular chamber is open to atmospheric pressure. Excised porcine MVs are mounted and attached to the MV annulus holder that separates the atrial and ventricular chambers. The negative atrial-chamber pressure establishes a pressure difference across the mounted MV and closes the valve in the same fashion that it closes with a pressurized ventricle; the fluid movement towards the atrium through the valve drives the leaflets to closure.

### Left Heart Model

The left heart model system (Fig. 1a) was constructed with 12.7 mm (0.5 in.) clear-cast acrylic sheet. Acrylic sheets were machined to size, and the pieces were connected with bolts threaded into drilled and tapped holes. The inside edges were sealed with room-temperature-vulcanizing silicon to ensure the chamber was vacuum tight. To simulate the left atrium and left ventricle, the left heart model has two separate chambers. Physiologically, the ventricle is positioned inferior to the atrium; these positions were inverted in the left heart model to facilitate sub-valvular studies at peak pressure load. This inverted position leads to gravitational forces being exerted on leaflets and CT in the superior direction. However, during peak-load valve studies (for which the apparatus was intended), these unphysiological gravitational forces are negligible relative to the pressure force exerted on the valve. Previous dynamic systems have also neglected this effect from gravitational forces while providing valuable results [13, 14, 18].

The atrial chamber is within the acrylic walls, while the ventricular is above the partition plate, allowing the ventricular side of the valve to remain accessible during valve closure. This improves access to the ventricular side of the valve and the subvalvular apparatus. The pressure on the ventricular side of the valve is slightly higher than atmospheric due to the presence of a small amount of water, which simulates surrounding fluids and prevents tissue desiccation. It should be noted that 1 cm of water equals 0.74 mmHg, and by using a refill system (Fig. 1), the water line is kept at 5 cm above the annulus, covering the MV. Hence, the pressure difference across the MV leaflet membrane is the atrial side’s negative pressure relativeto atmospheric pressure plus 5 cm water (equaling 3.7 mmHg). The resulting hydrostatic force exerted on the tissue structures is diminishingly small compared to the forces on the MV apparatus from the trans-membrane pressure difference.

Figure 1a identifies the integral components of the left heart model. The ventricular/atrial orifice (i), located in the center of the partition plate, connects the two chambers. The excised MV is mounted directly above the ventricular/atrial orifice. When the system is filled with water, the ventricular/atrial orifice allows water to flow between the chambers to simulate backward blood flow through a leaking valve. The water level on the ventricular side is maintained at a constant height by a continuous refill system in which water is supplied into the testing chamber (Fig. 1). Excess water is permitted to drain through an overflow port, thereby keeping the water level on the ventricular side of the system constant. Maintenance of a stable water level ensures the small hydrostatic pressure against the ventricular side of the valve is constant and prevents desiccation of the CT and leaflets. The vacuum system input (ii) is attached to the vacuum system with a tube to allow for the establishment of negative pressure in the atrial chamber. The vacuum gauge input (iii) has a T-adapter attached. A digital vacuum gauge (DPGA-00; Dwyer, Michigan City, Indiana) and a pressure catheter (Mikro-Tip SPC-350; Millar Instruments, Houston, Texas) are connected to monitor and record the pressure in the atrial chamber. The vacuum release valve (iv) closes to seal the chamber and opens to restore the chamber to atmospheric pressure. The drain valve (v) closes to seal the chamber and opens to remove water from the chamber. It also attaches to a water source with a tube to fill the chamber.

### Excision and Mounting of Mitral Valves

**Fig. 2:**
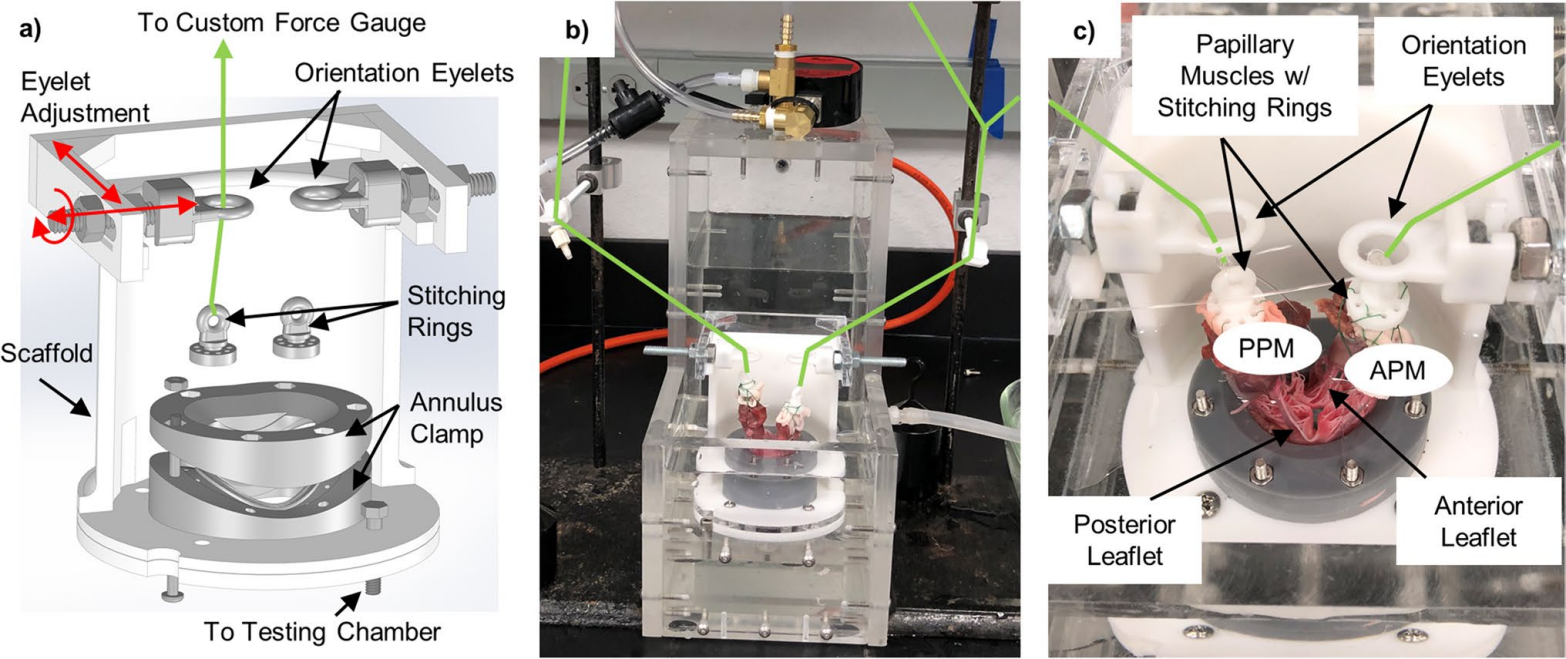
Valve mounting hardware and the scaffold shown in **a** exploded view and **b** and **c** with the scaffold mounted to the left heart model system. The scaffold positions the annulus clamp and PM orientation eyelets relative to each other and allows for attachment to the system. Green arrow denotes location of force transmission line tethering the papillary muscles through several orientation eyelets upwards to a pair of digital scales, fitted to measure PM force with 0.01N accuracy. APM and PPM: anterolateral and posteromedial papillary muscles, respectively

Porcine hearts were obtained from a local abattoir. Each MV was removed with the leaflets, annulus, PMs, and CT intact using the following procedure. The heart was positioned with the anterior portion pointing upward, and the left atrial appendage was removed with scissors. The left thumb was inserted into the aorta as deeply as possible without damaging the heart. This created a barrier to prevent damage to the MV leaflets, PMs, and CT. The bottom prong of the scissors was inserted into the aorta until it rested on the thumb at the opposite side of the MV. The upper prong was over the outside of the aorta, and the heart was cut down the sagittal plane to the apex of the heart. This opened the heart to show the left and right atrioventricular junctions, providing a clear view of the MV. The PMs were removed from the left ventricular myocardium, and the valve was detached by cutting into the atrial and ventricular myocardium 5–10 mm from the annulus. From this point, the PMs were trimmed to create a flat edge approximately 9–10 mm below the average location of all chordae tendineae insertion points. The valves were trimmed to leave approximately 1–2 mm of myocardium around the annulus. The excised MV and PMs were then secured to the mounting system shown in Fig. 2.

The hardware used to mount the valves at the annulus and PM anchoring points (Fig. 2a) was developed in-house and has been previously described [11]. A two-piece clamp holds the annulus and locks onto the remaining myocardium, preventing valve slippage with small, inward-facing teeth. The clamp’s shape ensures the annulus adopts its physiological systolic, saddle-shaped geometry. The flat, bottom edge of each PM is loosely wrapped with polyester/dacron for suture anchoring and sutured to a stitching ring. The stitching rings have an eyelet by which they attach to an inextensible line (Fig. 2b and c). This line is routed through the PM orientation eyelets and connects to a pressure plate, ultimately transmitting the PM force to the scales. The annulus clamp and all components of the PM holder were 3D printed using a Form 2 printer (Formlabs, Somerville, Massachusetts) using standard Formlabs Grey resin at a 50-micron resolution. The mounting hardware is capable of being designed to be customized to each valve’s specific annulus and PM geometry using in vivo echocardiography measurements prior to animal euthenization. However, the hardware employed in this study was created using average values to focus on demonstrating feasibility of the system.

The annulus clamp and PM orientation eyelets were secured to a scaffold (Fig. 2a) to correctly position and orient the PM relative to the valve. The scaffold is constructed such that the PM eyelet positions can be easily adjusted to alter the PM placement. Once the valve was fully mounted, the scaffold was attached to the testing chamber. The bottom plate of the scaffold bolts to the partition plate on the testing chamber. A thin rubber gasket is placed between the scaffold and testing chamber to prevent leakage around the mounting system. The leaflets were positioned immediately above the ventricular/atrial orifice. Figure 2b and c show a mounted and attached valve.

### Testing Procedure

Once a MV had been mounted and attached, the testing chamber was filled via the drain valve until the MV was completely submerged. The testing chamber was then sealed by closing the drain and vacuum release valves. The vacuum system was then used to establish a pressure difference.

The vacuum system consists of an 11.35 L (three gallon) intermediary vacuum chamber and vacuum pump (BVV VE115, Naperville, Illinois). The vacuum chamber was connected between the pump and the testing chamber. The vacuum pump employed here can only remove air at a single, fixed rate. The intermediary vacuum chamber was therefore included to provide increased control over the establishment of negative pressure. This was achieved by incorporating a regulating valve that can be opened to varying degrees so as to more precisely control the atrial-chamber pressure. The intermediary vacuum chamber has two ball valves: the regulating valve and a charging valve. The charging valve was connected to the vacuum pump, and the regulating valve was connected to the testing chamber. With the regulating valve closed and the charging valve open, the pump creates negative pressure within the intermediary chamber without altering the pressure within the testing chamber. Once the desired intermediary chamber pressure had been reached, the charging valve was closed. At this point, the intermediary chamber can maintain the vacuum until needed. When the regulating valve was opened, the vacuum within the intermediary chamber pulled air from the testing chamber, establishing negative pressure within the atrial-chamber airspace. This negative pressure transmits through the water in the testing chamber to establish a pressure difference across the mounted valve.

After establishing negative pressure with the vacuum system, the regulating valve was closed when the airspace pressure was approximately − 120 mmHg to simulate systolic pressure differences in the left heart across the MV. As the water flowed through the ventricular/atrial orifice in response to the pressure difference, the mounted MV closed. The fluid flow required to close the valve, as well as any subsequent backflow if the valve was having any regurgitant flow, was reflected in a change in atrial chamber’s fluid level (Fig. 1c), as well as the difference between water supply and drainage return rates in the ventricular side. When the vacuum release valve was opened, the atrial-chamber airspace returned to atmospheric pressure, eliminating the pressure difference. The MV opened, and water flowed back into the ventricular chamber from the atrial chamber. Care was taken to ensure the PMs and CT remain wet during closure to prevent desiccation.

**Fig. 3:**
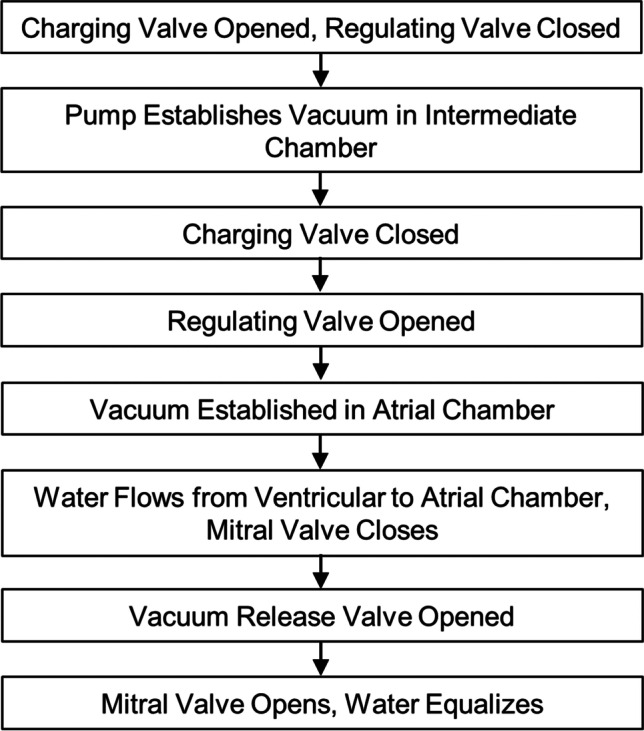
Flowchart representation of vacuum system operation during testing procedure

The water was not replaced between subsequent tests of the same mounted valve as the levels equalize when vacuum pressure is removed. The testing chamber was elevated and a digital camera was placed beneath the atrial chamber, oriented upward toward the leaflets. Still images were taken of the closed valve from the ventricular and atrial side to document coaptation of the leaflets. The testing procedure is summarized in Fig. 3.

### Measurement of Trans-Valvular Pressure Differences and Papillary Muscle Forces

To demonstrate the utility of the apparatus, PM forces were measured using a pair of digital scales customized to operate as force gauges. Each PM was connected to a scale through a force bar via a section of high-strength, inextensible line. The lines were secured to the muscles by stitching rings, then passed through several positionable eyelets such that the position and orientation of each muscle was physiologically realistic, and finally attached to the force gauge. The PMs were manually positioned such that all CT were taught when the valve closed. Valves were repeatedly closed after mounted in the system to ensure the tissue was preconditioned prior to all data collection.

The negative pressure in the atrial-chamber airspace approximates the pressure differences across mounted MVs. The vacuum gauge was used to monitor the atrial chamber pressure while the pressure catheter was used to record the exact pressure during valve closure tests. The pressure catheter was connected to a controlling unit (PCU-2000; Millar Instruments, Houston, Texas) via a PEC-10D cable (Millar, Inc.). Data acquisition was achieved with a digital oscilloscope (Siglent SDS 1202X-E; Siglent Technologies, Solon, OH) via a phono-to-BNC cable. Data acquisition was initiated by internally triggering against a falling edge. Oscilloscope data was downloaded to a USB flash drive for further analysis.

To calibrate and convert the data from voltage to pressure, multiple pressures while the valve was fully closed from approximately − 10 mmHg to − 160 mmHg (determined with the vacuum gauge) were measured with the pressure catheter and recorded on the oscilloscope. A cubic fit was performed on the calibration data, and the equation of best fit was obtained. This equation related oscilloscope signal voltage to pressure and was used to convert all subsequent voltage measurements.

### Measurement of Chordal Forces

Chordal force was measured in a single secondary (strut) chord using a custom developed force transducer (Fig. 6). The force gauge utilizes a specially designed cantilever frame for chordal studies and is configured for a full Wheatstone bridge strain gauge measurement for optimal signal-to-noise ratio. The frame attaches to the chord at two points via silk suture before the chord is cut in between, leaving the frame “bridging” the cut chord. The details of this gauge, including the design and construction, are outside the scope of this study and will be presented in a future publication. Data from this force gauge was acquired with a NI (National Instruments, Austin, Texas) cDAQ 9174 acquisition chassis with a NI 9237 strain gauge input module through a custom VI.

### Quantification of Regurgitant Flow Rate

Water movement from the ventricular side to the atrial chamber when the valve is closed is quantified as regurgitant flow. A degree of backflow through the valve could be observed by the continuously decreasing water level in the ventricular chamber during closed valve tests. If employing a refill system to keep ventricular water level constant, the changing level of the atrial chamber may be used to measure backflow. This may be done electronically via level sensors, optically with a camera and vision software, measuring the difference in refill and overflow return flow rates, or simply by noting the total level change. To evaluate the average flow rate, a mark was placed on the outside of the ventricular chamber prior to MV closure. A valve closure test was then performed and the final height marked before the vacuum release valve was opened. A stopwatch was used to record the time between establishment of negative pressure and marking of final water height. The change in height of the water was measured and recorded. The average flowrate is taken to be the change in volume (determined from the height change and the known cross sectional area of the system) divided by the measured time. This procedure was repeated three times and the results averaged.

To determine if any inter-hardware leaks were present, the porcine valve was replaced by a solid piece of rubber, mimicking a completely sealed valve. The mounting hardware was reattached to the testing chamber, and the initial water level was marked. Negative pressure was then established and maintained for 2 min. The flowrate through the valve mounting system was calculated and subtracted from the through-valve flowrates.

## Results

**Fig. 4:**
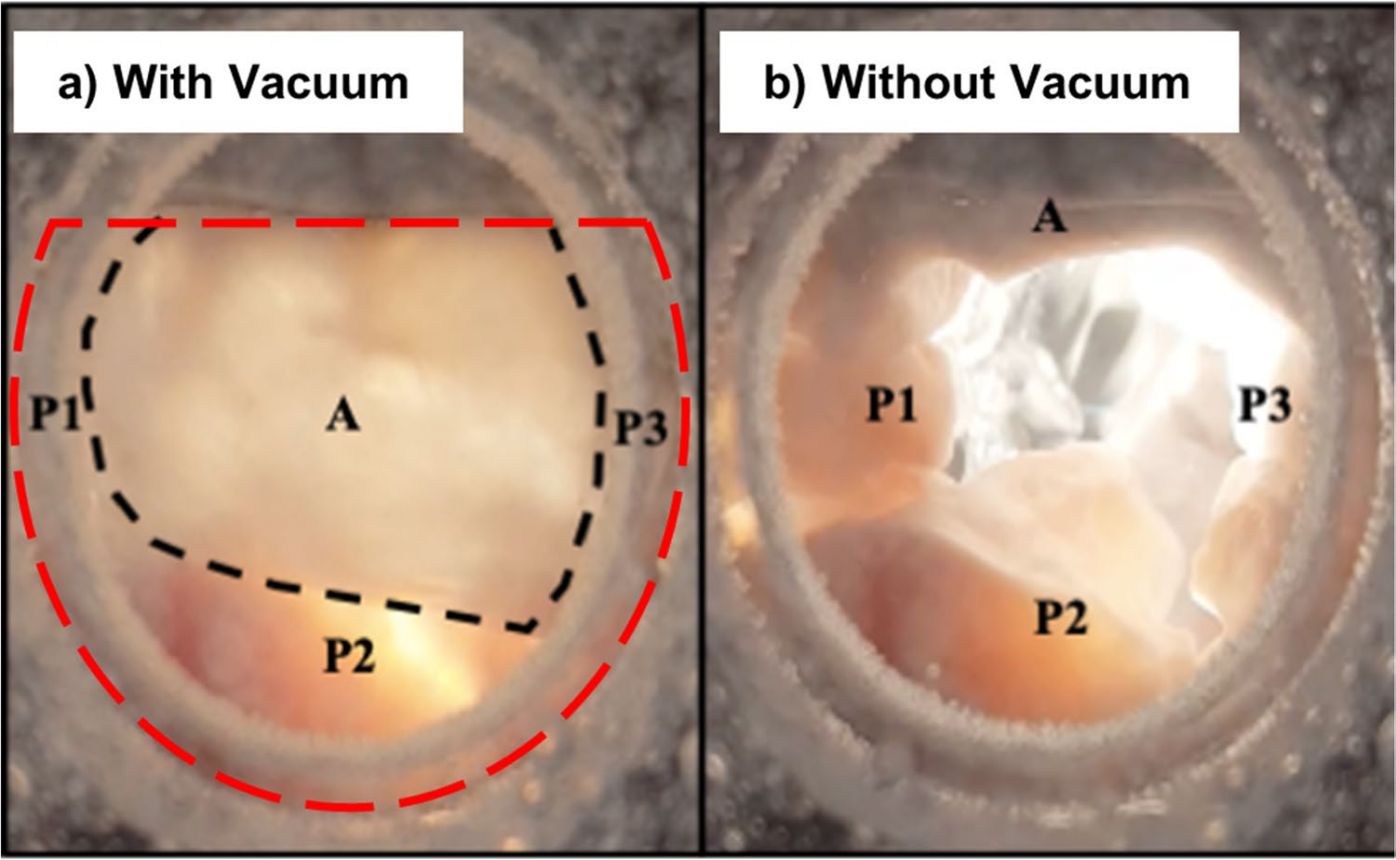
Atrial view of a mitral valve during a closure test. With the testing chamber elevated, still images were taken displaying the valve through the atrial chamber. The anterior leaflet (A) and regions of the posterior leaflet (P1, P2, and P3) are labeled and the coaptation line is marked with the dotted black line. **a** valve fully closed, the anterior leaflet can be seen covering most of the orifice; **b** after releasing the vacuum, the valve re-opened as the pressure difference was eliminated. The characteristic “D-shape” of the mitral valve annulus outline is denoted by the dotted red line of the closed valve

Example images from static-pressure closed valve tests are shown in Fig. 4. In each test, the anterior and posterior leaflets reached coaptation that was concave to the ventricular chamber, with the anterior leaflet covering most of the annular orifice area. This resembles physiological valve closure in vivo.

### Measurement of Papillary Muscle Forces

**Fig. 5:**
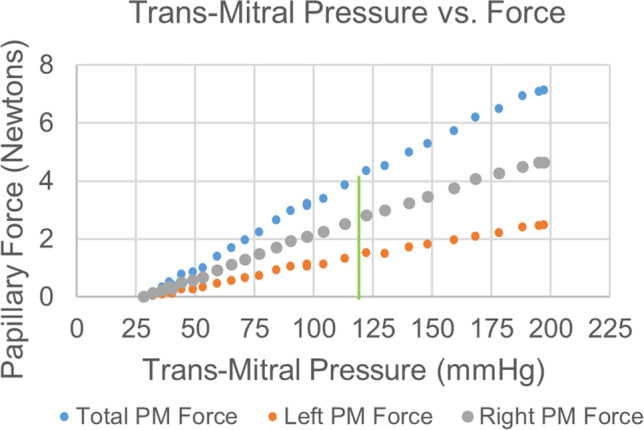
Individual and total papillary muscle force as a function of trans-mitral pressure difference. Green line denotes physiological trans-mitral pressure of 120 mmHg

Figure 5 shows the total PM force, computed from the sum of the two PM forces, as a function of trans-mitral pressure on the MV leaflets. The total PM force varies linearly with pressure within an approximately physiological pressure range, having an *R*^2^ value of 0.9963. Total papillary force at 120 mmHg transmitral pressure was found to be 4.07 N. The upper set of orientation eyelets was not used in collecting this dataset.

### Measurement of Chordal Forces

**Fig. 6:**
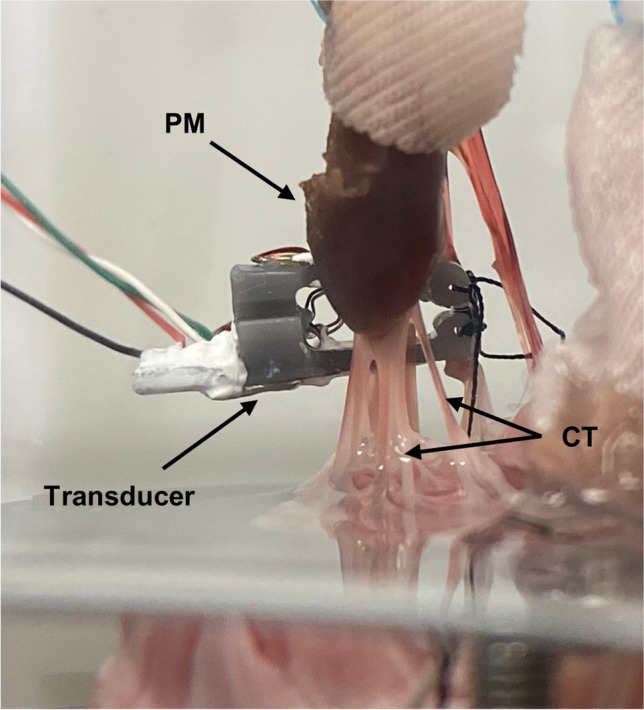
Custom built full Wheatstone bridge based chordal force transducer on secondary strut chord

Figure 6 shows the custom built chordal force transducer in place, sutured onto and bridging a secondary strut chord. A maximum exemplary chordal force of 0.285 N was measured.

### Quantification of Regurgitant Flow Rate

Representative regurgitant flow rates through the MV is shown in Table 1 from three test valves and a reference rubber cap that seals the annular area. As demonstrated, water may travel from the ventricular to the atrial side of the testing chamber not only through the valve, but also in small, insignificant amounts through/around the mounting system. The average regurgitant flowrate, with inter-hardware leaks removed, was 12.1 ± 0.51 ml/s.

**Table 1:** Backflow (regurgitant) flow rates through mounted porcine mitral valves. Flow rates were calculated from the change in water level divided by closure time. Backflow through the mounting system itself was measured by replacing the valve with an impermeable section of rubber

	MV 1	MV 2	MV 3	Rubber
ΔHeight (mm)	13.5	13.1	13.5	8.6
Regurgitant rate (mL/s)	13.7	12.5	12.8	0.90

## Discussion

Having access to the ventricular side of the atrioventricular valves during in vitro testing has been demonstrated to improve access and simplify measurements on the subvalvular structures. This will enable much improved access and quality for any procedures and measurements on these structures in future experiments. The testing system repeatedly kept the MVs closed and prevented tissue dessication. Leaflet coaptation that resemble those found physiologically and from previously dynamic systems was observed. Previous in vitro testing systems (using positive ventricular pressure) have been proven to mimic animal models’ MV mechanics during systole [19]. Our results support the feasibility of using negative pressure to replicate in vivo MV closure in a peak-load configuration. The most important advantage of the testing chamber is the access it provides to the ventricular side of the subvalvular apparatus of the MV, the PMs, and the CT. This is due to the inversion of the ventricular and atrial chamber positions and the use of negative pressure.

Prior in vitro and in vivo studies have shown PM force to be on the order of 7–8 N [13, 20, 21]. Several features of the new vacuum-based left heart model contribute to the comparatively lower PM forces measured. Firstly, this system mounts the mitral annulus in a physiologically realistic saddle-shape, rather than along a flat plane. Not only does this more closely mimic the native shape of the annular anchoring, but it also significantly reduces the overall annular stress [2, 4, 22]. Also, the method of PM positioning employed, in addition to making it quick and straightforward to adjust, leaves the PMs free to reposition and/or reorient due to their non-rigid attachment. PM displacement has been shown to strongly affect overall valve competence and CT force distribution [23, 24]. Many in vitro experiments utilize rigid PM mounting, whereby the PMs are inflexibly fixed in 3D space. Without conducting echocardiographic measurements, the native PM locations cannot be known, meaning the experimentally mounted PMs are likely displaced somewhat from their native, stress minimizing positions. By allowing the PMs to move into an optimal 3D position and angle, they are permitted to arrange themselves into a stress minimizing orientation, reducing PM force.

The orientation eyelets employed were effective at redirecting the lines from the PMs upwards to the scales. Care should be taken to eliminate/minimize the friction between the lines and the eyelets, otherwise undesirable effects such as stiction may occur. This may be accomplished by a variety of means such as eliminating sharp edges on the eyelets, using smooth, slick line, or by adding a lubricant. More complex solutions may utilize bearing mounted guide wheels.

For the work presented here, atrial pressure was measured through the port at the top of the testing chamber. Measurements have been performed using the same equipment but with the catheter’s pressure-sensing tip located immediately below the closed valve (on the atrial side), and the measurements were found to be within 5% of those obtained from the top port. The ventricular hydrostatic pressure is maintained constant by the refill system, while the atrial pressure is controlled by the pre-pressurized intermediate vacuum chamber. Some gradual depletion of this vacuum pressure was observed (approximately 1 mmHg/s), mostly due to valve leakage. This depletion is, however, sufficiently slow to easily permit many measurements before the trans-mitral pressure drops below physiological levels. Alterations in the size or location of the vacuum port may influence atrial pressure, but the similarity of pressures measured in the vacuum airspace vs. immediately below the valve indicate that this effect is diminishingly small (difference consistently measured to be less than 5%). If additional control is necessary, a secondary vacuum chamber may be placed in parallel to replenish the vacuum. Minimization of the atrial chamber vacuum airspace and/or water may permit faster vacuum establishment, though this comes at the cost of decreased regurgitant volume before fluid reaches the vacuum port. Additionally, it is possible to simply normalize to account for any minor pressure fluctuations in post-experimental data analysis.

Access and visibility provide opportunities for many types of in vitro MV experiments, such as leaflet stability, valve competency, and surgical repair techniques. In particular, force measurements in the PMs and chordae tendineae are important for understanding MV mechanics [22, 25]. Force measurement experiments could be performed in this system without the restrictions of a sealed, pressurized ventricular chamber. This improves overall access to the valve and important structures, and could eliminate the need for additional calibration of force transducers due to pressure effects. While the PM and chordal force measurements presented are certainly possible with a sealed ventricular chamber, they are, based upon prior experience, necessarily more complex to perform [11, 13]. In addition to simplifying many measurements, unrestricted ventricular access also enables previously difficult/impossible investigation methods, such as optical tracking of fiducial markers on the sub-valvular apparatus. Finally, the system and experiments detailed above are part of an overall strategy of improving access of experimental data to computational models [9, 26, 27].

## Limitations

Regurgitant flow, indicating valve leakage, was present to some degree in all valve closure tests. The data in Table 1 indicates that the water flow through the valve leaflets was substantially greater (> 10X) than flow through the mounting hardware. Clinically, mild mitral regurgitation may be classified as being upwards of 20% of stroke volume [28]. Given the typical at rest cardiac output for an adult of 5–6 L/min, this corresponds to approximately 1.5 L/min of regurgitant flow; our measured in vitro regurgitant flows were smaller than this.

Backflow through the valve leaflets could have multiple causes. Firstly, some regurgitation can be considered normal. Healthy pigs used as controls for studies commonly have small amounts of mitral regurgitation [29]. Secondly, the valves might have been dysfunctional in the live animals. Methods such as echocardiography are commonly used to assess mitral regurgitation in vivo [30]. We could not determine if regurgitation was present in our live animals since the hearts were retrieved postmortem. In vivo comparisons of regurgitation could also be used to explore the system’s ability to simulate in vivo systolic valve function. Similar backward flow in vivo and in the system would provide support for accurate simulation [19]. Finally, disrupting the physiological structure of the MV apparatus may cause regurgitant flow. The mounting hardware used in our tests was constructed based on average measurements of porcine mitral valves, not tailored to each valve, which would require in vivo echocardiographic measurements [31].

As a final note regarding regurgitant flow, water should not be permitted to enter the intermediary vacuum chamber. The system was designed to accommodate backflow by placing the vacuum system input near the top of the atrial chamber. At the vacuum system input’s current height, the atrial chamber can tolerate an influx of approximately 2 L of water throughout a valve closure test.

## Conclusions

During in vitro experiments with the mitral valve, sealing and pressurizing the ventricular chamber inhibits access to important components of the mitral valve apparatus. We developed a testing system that closes excised mitral valves with negative pressure on the atrial side, leaving the ventricular side open to atmospheric pressure. In the system, the leaflets formed coaptations that resembled valve closure in vivo, with concave geometry relative to the ventricular chamber and the anterior leaflet covering the majority of the ventricular/atrial orifice. PM and chordal force measurements were performed to demonstrate the utility of greatly improved ventricular access in a peak-force setup. Throughout the tests, access to the valve leaflets, chordae tendineae, and PMs was maintained. This technique provides new ways to experiment with and enhance understanding of the mitral valve.

## Abbreviations

MV: Mitral valve
PM: Papillary muscle
CT: Chordae tendineae

## Declarations

### Animal Studies

No animal studies were carried out by the authors for this article.
